# Comparison of two asymmetric headgear force systems: A finite element analysis

**DOI:** 10.1590/2177-6709.24.2.41.e1-6.onl

**Published:** 2019

**Authors:** Samaneh Sadeghi, Zohreh Hedayati, Batoolalsadat Mousavi-Fard

**Affiliations:** 1 Kerman University of Medical Sciences, Kerman Dental School, Department of Orthodontics (Kerman, Iran).; 2 Shiraz University of Medical Sciences, Shiraz Dental School, Department of Orthodontics (Shiraz, Iran).; 3 Afzalipour Hospital, Kerman University of Medical Sciences (Kerman, Iran).

**Keywords:** Asymmetric, Extraoral traction appliances, Finite element analysis

## Abstract

**Objective:**

The aim of this study was to evaluate the effect of displacement patterns of the molar teeth in response to different asymmetric headgear loading using 3D finite element method.

**Methods:**

A series of twenty-five facebow with different left *vs*. right outer bow length and different expansion of left *vs*. right were designed. The non-favored side (right side) was shortened at intervals of 10 mm, and favored side (left side) was expanded 10 degree greater than right side and 5 degree expansion were successively added. At the first phase, each side received 200-g load, implying the neck strap to displace toward shorter arm. At the second phase, a total of 400-g load was applied to the ends of the outer bow. Because of the neck strap displacement, the shorter arm received greater load than the left side, the magnitude of the applied force to each side depended on difference of left *vs*. right outer bow length and expansion.

**Results:**

All systems were effective in promoting asymmetric distal movement of the molars. However, the asymmetrical facebow with the 40 mm shortening and 25 degree expansion outer bow when unequal force applied could be used in asymmetric mechanics. Medial and occlusal displacing forces were observed in all systems.

**Conclusions:**

Both equal and unequal force application is effective for molar distalization. Expansion of the outer bow in the affected side and shortening of the outer bow in the normal side were effective to produced differential distal molar movement.

## Resumo

**Objetivo:**

o objetivo desse estudo foi usar o método de elementos finitos 3D para avaliar o efeito no padrão de deslocamento dos molares em resposta ao uso de aparelhos extrabucais com diferentes forças assimétricas.

**Métodos:**

foram confeccionados 25 aparelhos extrabucais (AEB) com diferenças, entre os lados direito e esquerdo, quanto ao comprimento e ao grau de abertura do braço externo. O lado não favorecido (lado direito) foi encurtado em intervalos de 10 mm e o lado favorecido (lado esquerdo) foi aberto 10 graus a mais do que o lado direito, sendo adicionados 5 graus de abertura sucessivamente. Na primeira fase, cada lado recebeu carga de 200 g, causando o deslocamento da tala cervical em direção ao braço mais curto. Na segunda fase, foi aplicada carga total de 400 g às extremidades dos braços externos dos AEB. Devido ao deslocamento da tala cervical, o braço mais curto recebeu uma força maior do que o lado oposto; a magnitude da força aplicada em cada lado dependeu da diferença no comprimento e na abertura dos braços externos do AEB.

**Resultados:**

todos os sistemas foram efetivos em promover movimentação distal assimétrica dos molares. Porém, o AEB assimétrico com 40 mm de encurtamento e 25 graus de abertura do braço externo poderia ser usado na mecânica assimétrica, com aplicação diferenciada de força. Foram observadas forças mediais e oclusais de deslocamento em todos os sistemas.

**Conclusões:**

tanto a aplicação de forças simétricas quanto assimétricas são efetivas para a distalização dos molares. A expansão do braço externo do AEB no lado afetado e o encurtamento no lado normal foram efetivos na produção de movimento distal assimétrico dos molares.

## INTRODUCTION

Headgear was introduced at the first time in early 1800s, until now, different modifications have been used.^1^ It could be used to restrain maxillary growth, retract maxillary molars, or hold the molars in place to reinforce the anchorage while retract canine and incisors^2^. However, to obtain successful results, extraoral traction requires considerable patient compliance. Different treatment modalities have been introduced to distalize maxillary molars to overcome patient compliance, such as palatal bar, repelling magnets, Nitinol coil spring, K-loops, superelastic wires, Wilson arches, Jones jig appliances, pendulum appliances, distal jet appliances and recently temporary anchorage devices (TAD).^1^ Compared with these appliances, headgear is the better choice because of restricting effect of maxillary growth and dental movement to correct Class II relationship.^3^ In some instances, there is unilateral Class II malocclusion (Class II subdivision), in which one side presents Class II molar relationship, while the other side is Class I. Such situation requires an asymmetric force system. Some changes in facebow convert symmetric headgear into asymmetric ones. These changes include asymmetric length of the right/left outer bow, which are referred as power arm facebow, asymmetric length of the right/left inner bow, different angulation right/left between inner and outer bow, swivel offset, hinged inner bow, different toe-in bend in the inner bow, or combination of them^2^. The most practical method to design asymmetric headgear is shortening one outer bow or elongating one inner bow^2^. Extensive clinical data have revealed the effectiveness of asymmetric headgear in unilateral distalization^4^. For better understanding of dental biomechanical behavior, the finite element analysis (FEA) was introduced in 1973 and is a useful method to quantify forces, moments and tensions, as well as other variables that allow appliance activations to be simulated for distal movement according to coordinates X, Y and Z. It is based on the separation of the analysis shape into subdomains through finite elements that could predict the mechanical behavior of the object under varied loading conditions.^5^ ^,^ ^6^

Despite the existence of a number of investigation on the biomechanics of unilateral facebows, there is still conflicting concepts regarding their effects. Nobel and Waters^7^ found that asymmetric headgear produced a buccal displacement in the transverse dimension as a side effect. However, in a study by Hershey et al,^8^ some buccal-buccal displacement and some lingual-buccal displacement of the molars was found. The buccal-buccal displacement was attributed to the arch expansion effect of the inner bow.^8^ However, until now there is no study regarding the effect of the neck strap displacement and unequal force application on the molar movement. Thus, te objectives of the present study were:

1. To evaluate the effect of neck strap displacement on differential molar distal, lateral and extrusive forces.
2. To assess the 3D molar displacement with respect to differential shortening of one side of the outer bow in relation to progressive increases in the difference of the length of outer bow.
3. To assess the 3D molar displacement while expanding one side of the outer bow in relation to progressive increase in expansion.
4. To evaluate the more effective system on molar distalization, either the asymmetric outer bow shortening or the asymmetric outer bow expansion.

## MATERIAL AND METHODS

**Figure 1:**
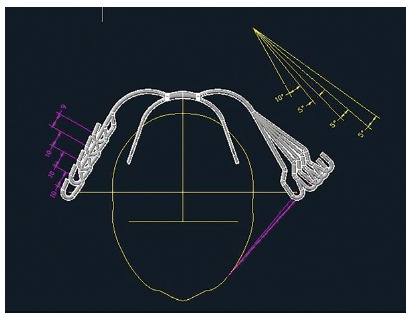
Geometry variation of symmetrical and asymmetrical face bows used in present study.

The asymmetric headgears were designed by the same operator based on measurements made from a commercially available facebow (Ortho Technology, Inc. Tampa, Florida, USA). The values attributed for characterizing the facebow behavior made of stainless steel were 200 GPa for the modulus of elasticity and 0.3 for Poisson's coefficient. Outer bow was considered to have elastic deformation when applied distalization force. Boundary conditions were assigned to the nodes at the end of the inner bows, where the inner bow insert to headgear tubes, as zero displacement perpendicular to inner bows. The facebow was assumed to be homogeneous, isotropic, and linearly elastic. The facebows were created through ANSYS software, version 12.0.1 (Swanson Analysis System, Canonsburg, PA). A total number of 256,611 nodes and 127,978 brick elements of hexahedral and tetrahedral solid element were used to construct the facebow. The nodes at the end of each inner bow sides consisted of 3D coordinates (X, mediolateral direction; Y, Anteroposterior displacement; and Z, supero-inferior direction) and their boundary conditions. A series of 25 facebows with different left *vs*. right outer bow length and different expansion of left *vs*. right were designed. The right side was shortened at intervals of 10 mm and left side was expanded 10 degrees greater than right side, and 5 degrees expansion were successively added (Fig 1). The initial outer bow length was 72 mm. Movement toward the lateral side, extrusive displacements and distal side were considered to have negative sign, while other movements considered having positive sign. Nodes at each end of inner bow were used to assess the displacement. At this phase of the study, two different loading modes were applied at the ends of the outer bow.

At the first phase, each side received 200-g load, implying the neck strap to displace toward shorter arm (right side). At the second phase, a total of 400-g load was applied to the ends of the outer bow. In essence, since the neck strap does not displace, the right side received greater load than the left side, the magnitude of the applied force to each side depended on difference of left *vs*. right outer bow length and expansion. The direction of facebow relative to true horizontal plan was at 9.5 degrees angulation (Fig 1).

## RESULTS

All the results were divided into two parts. The first part consisted of the evaluation of molar movement when the applied loads were similar in left and right side, and the second part consisted of the evaluation of various load applied to left *vs*. right side (when the neck strap was not displaced).

### Part one: equal force

**Figure 2:**
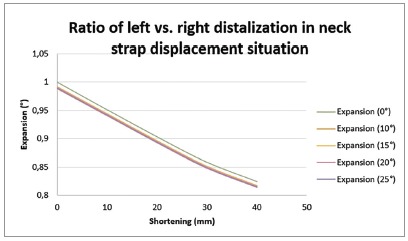
Ratio of left *vs*. right distalization in neck strap displacement situation: (+) left *vs*. right mesiodistal movement was not in the same direction.

*» Anteroposterior displacement -* The results revealed that the affected side (left side) underwent greater movement toward posterior than right side (Fig 2). The amount of distalization increased with an increase in outer bow length difference. With an increase in left *vs*. right outer bow expansion difference, the differential molar distalization was increased.

**Figure 3:**
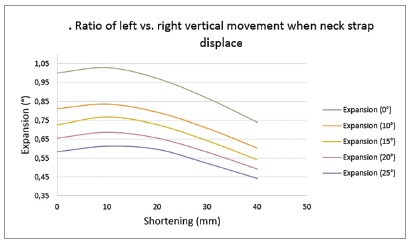
Ratio of left *vs*. right vertical movement when neck strap displace: (+) left *vs*. right vertical movement was in the same direction.

»*Mediolateral displacement* - Posterior segment in the affected and unaffected sides underwent palatal tipping. The amount of buccal tipping decreased in accordance to the outer bow difference increase, as can be seen in Table 1.
»*Supero-inferior displacement* - These finding showed extrusion of the posterior teeth of normal side and affected side. With the increase in the expansion of the outer bow, the supero-inferior ratio of left *vs*. right side decreased. With an increase in the outer bow length difference, the supero-inferior ratio of left *vs*. right side increased (Fig 3).

**Tabela 1:** Ratio of left vs. right Bucco-lingual movement when neck strap displace.

Expansion (degrees) / Shortening (mm)	0	10	15	20	25
0	-1.000	-0.992	-0.989	-0.989	-0.990
10	-0.951	-0.944	-0.941	-0.941	-0.942
20	-0.903	-0.896	-0.893	-0.893	-0.894
30	-0.858	-0.851	-0.848	-0.848	-0.849
40	-0.824	-0.817	-0.815	-0.814	-0.815

(+) left *vs*. right bucco-lingual movement was not in the same direction; (-) left *vs*. right bucco-lingual movement was in the same direction.

### Part two: unequal force

**Figure 4:**
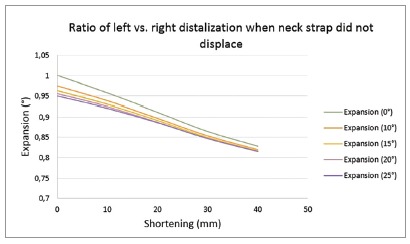
Ratio of left *vs*. right distalization when neck strap did not displace: (+)left *vs*. right mesiodistal movement was not in the same direction.

*» Anteroposterior displacement* - In this situation, the affected side revealed greater posterior movement, compared with previous situation. As the left*vs*. right outer bow length and expansion difference increased, the differential molar distalization increased (Fig 4).

**Figure 5:**
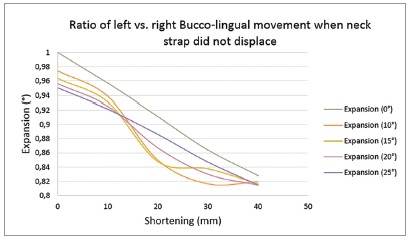
Ratio of left *vs*. right bucco-lingual movement when neck strap did not displace: (+) left *vs*. right bucco-lingual movement was not in the same direction; (-) left vs. right bucco-lingual movement was in the same direction.

*» Mediolateral displacement* - It was found that neck strap fixation showed similar results to neck strap displacement (Fig 5).

**Figure 6:**
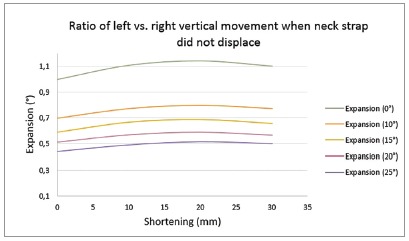
Ratio of left *vs*. right vertical movement when neck strap did not displace: (+) left *vs*. right vertical movement was in the same direction; (-) left *vs*. right vertical movement was not in the same direction.

*» Supero-inferior displacement* - With the exception of the 25 degree expansion in addition to a 30-mm shortening in the right side configuration, vertical displacement of posterior teeth in this situation was approximately similar to neck strap displacement situation. The posterior teeth underwent palatal tipping, with apexes tending to move away from midline because of the changes in axial inclination (Fig 6).

## DISCUSSION

Alternative intraoral molar distalization modalities have been introduced to overcome patient noncompliance. Because of the inevitable side effects of these appliances, it is suggested to be used before eruption of the second molar.^10^ Yet, headgear appliances can be used to retract the molar teeth after eruption of the second molar.

Studies on distalization usually are conducted on bilateral molar. Some authors have investigated the unilateral molar distalization with intra-arch appliances. When facebow is activated, not only distal force is applied to the molar, but also unwanted mediolateral and superoinferior forces are created. To the best of our knowledge, until now there is no published article regarding the effect of displacement of the neck strap or the effect of equal and unequal forces on the movement of the posterior teeth in the three-dimensional analysis.

Finite element analysis is proved to be effective in different applications in Orthodontics.^3^ Because experimental technique in human or animal are limited, finite element analysis is a good solution to simulate the effect of different appliances on the dental structures.

In relation to asymmetric distal movement of the molar, both equal and unequal force systems have shown to be effective, although when equal force is applied, the molar movement in affected side present greater magnitude. With expansion of outer bow in the left side, the ratio of the mesiodistal movement of the left to the right side in both equal and unequal force systems is decreased. Shortening of the outer bow in the normal side also leads to a smaller ratio of the left to the right mesiodistal movement, which indicates to greater displacement in the affected side. The greater the asymmetric bow, the greater the unilateral effectiveness of facebow will be. With the asymmetrical facebow, the favored molar receives a higher share of either distal force, equal force applied, or unequal force applied. The key point at producing asymmetric force is the facebow configuration. The greater the asymmetry of the facebow, the greater the share of the force applied at desired teeth.

Drenker et al^9^ stated that to produce unequal distally directed force, the favored side external bow should be expanded and lengthened. Further increase in the lateral displacement and lengthening would continue to increase distal force in the desired side. Lateral expansion about three fourths of an inch and lengthening of the outer bow about two inches on the desired side, compared to undesired side, results in unilateral action, in the average case.^9^

Geramy et al^10^ showed that as the degree of unilateral expansion increased, the amount of distal force on the expanded side also increased.

In both systems, palatal tipping of the right and left side are inevitable. The amount of this unwanted movement in the affected side increased with the expansion. Shortening the right side has a similar effect. This reaction tends to move the favored and the non-favored molar into lingual crossbite. This finding is not in accordance with Geramy et al,^10^ ^,^ ^11^ who showed that lateral force results in lingual crossbite in the intact bow side and buccal crossbite in the short bow side. The magnitude of this mediolateral force increase as the unilateral effectiveness of the facebow increase. This conclusion agrees with Nobel et al^7^ regarding the generation of undesirable mediolateral forces when an asymmetrical facebow is activated. The amount of lateral movement increased as the asymmetry of the outer bow was increased.^8^ To prevent this undesirable side effect, Hershey et al^8^ recommended that the long arm has to terminate posteriorly near the first molar and expanded laterally so that in an activated state, the traction strap converge toward the midsagital plane of the patient. The short arm also should terminate near the position of the canine tooth and extended laterally, to allow its tip to gently touch the soft tissue of the cheek and traction strap on that side, parallel to the patients midsagital plane^8^. Constriction and expansion of the inner bow also may be effective in contracting this unwanted side effect^12^. Angles inequality of left and right external arm result in lateral movement of teeth, which is undesirable, except in the case of crossbite correction. It seems that expansion of the longer bow could not overcome lingual crossbite tendency and, as Valrik and Iscan^13^ stated, expansion of the inner bow could be used in this situation. According to Yoshida et al,^14^ the amount of mediolateral movement increase as the asymmetry of the outer bow was increased. As Drenker^9^ expressed, one of the most common cause of unilateral action of symmetric facebow is the friction between the neck strap and neck, which offsets the equality applied force at the outer bows. Contrary to the results obtained in the current study, some authors have reported that buccal displacement occurred on both molars.^7^ ^,^ ^8^

Because of the cervical pull applied, posterior teeth in both systems undergo extrusive movement, the apexes tend to move toward buccal, and the crown tip toward palatal side in both sides. As expected in this study and previous studies,^1^ ^,^ ^15^ increasing the distalization displacement, extrusion of teeth in the affected side was increased and the first order and third order movements were increased. The correction of asymmetric molar extrusion should not be performed by adjustment of the facebow, but with a multibonded appliance after headgear therapy.

As Baldini^15^ described, archial expansion effect of an activated flexible bow culminates in increasing the intermolar width as the upper molar teeth moves distally. However, extrusive forces produce the moment that tend to tip the crown palatally. It seems that this moment overcomes the archial expansion effect, and as teeth move distally they tend to move in lingual crossbite.

## CONCLUSION

Based on the present FEM study, both equal and unequal force application are effective for bilateral unequal Class II relationship.

Expansion of the outer bow in the affected side was effective to produce differential molar movement, however, could not prevent from palatal tipping of the affected side teeth.

Shortening of the outer bow in the normal side also culminates in greater movement on the desirable side.

## References

[1] Altug H, Bengi AO, Akin E, Karacay S (2005). Dentofacial effects of asymmetric headgear and cervical headgear with removable plate on unilateral molar distalization. Angle Orthod.

[2] Brosh T, Portal S, Sarne O, Vardimon AD (2005). Unequal outer and inner bow configurations: comparing 2 asymmetric headgear systems. Am J Orthod Dentofacial Orthop.

[3] Squeff LR, Ruellas AC, Penedo ND, Elias CN, Sant&apos;anna EF, Casaccia GR (2009). Asymmetric headgear for differential molar movement a study using finite element analysis. J Orthod.

[4] Chi L, Cheng M, Hershey HG, Nguyen T, Ko CC (2012). Biomechanical reevaluation of orthodontic asymmetric headgear. Angle Orthod.

[5] Coimbra ME, Penedo ND, Gouvea JP, Elias CN, Araujo MTS, Coelho PG (2008). Mechanical testing and finite element analysis of orthodontic teardrop loop. Am J Orthod Dentofacial Orthop.

[6] Knop L, Gandini LG, Shintcovsk RL, Gandini MR (2015). Scientific use of the finite element method in Orthodontics. Dental Press J Orthod.

[7] Nobel PM, Waters NE (1992). Investigation into the behavior of symmetrically and asymmetrically activated face-bows. Am J Ortod Dentofacial Orthop.

[8] Hershey HG, Houghton CW, Burstone CJ (1981). Unilateral face-bows a theoretical and laboratory analysis. Am J Orthod.

[9] Drenker EW (1959). Unilateral cervical traction with a Kloehn extraoral mechanism. Angle Orthod.

[10] Geramy A, Martin D, Bouserhal J, Emadian Razavi ES, Hassanpour M (2016). Asymmetric head gear: a comparison between unilateral outer bow expansion and unilateral outer bow shortening - an energy approach using the finite element method. Chin J Dent Res.

[11] Geramy A, Hassanpour M, Emadian Razavi ES (2015). Asymmetric outer bowlength and cervical headgear force system 3D analysis using finite element method. J Dent (Tehran, Iran).

[12] Pirttiniemi P, Kantomaa T, Mantysaari R, Pykalainen A, Krusinskiene V, Laitala T (2005). The effects of early headgear treatment on dental arches and craniofacial morphology an 8 year report of a randomized study. Eur J Orthod.

[13] Varlik SK, Iscan HN (2008). The effects of cervical headgear with an expanded inner bow in the permanent dentition. Eur J Orthod.

[14] Yoshida N, Jost-Brinkmann PG, Miethke RR, Konig M, Yamada Y (1998). An experimental evaluation of effects and side effects of asymmetric face-bows in the light of in vivo measurements of initial tooth movements. Am J Orthod Dentofacial Orthop.

[15] Baldini G (1980). Unilateral headgear lateral forces as unavoidable side effects. Am J Orthod.

